# Charring temperatures are driven by the fuel types burned in a peatland wildfire

**DOI:** 10.3389/fpls.2014.00714

**Published:** 2014-12-16

**Authors:** Victoria A. Hudspith, Claire M. Belcher, Jonathan M. Yearsley

**Affiliations:** ^1^PalaeoFire Lab, Hatherly Laboratories, Department of Geography, University of ExeterDevon, UK; ^2^School of Biology and Environmental Science, University College DublinDublin, Ireland; ^3^Earth Institute, University College DublinDublin, Ireland

**Keywords:** charcoal reflectance, wildfire, All Saints Bog, Ireland, burn severity, pyrolysis intensity

## Abstract

Peatlands represent a globally important carbon store; however, the human exploitation of this ecosystem is increasing both the frequency and severity of fires on drained peatlands. Yet, the interactions between the hydrological conditions (ecotopes), the fuel types being burned, the burn severity, and the charring temperatures (pyrolysis intensity) remain poorly understood. Here we present a post-burn assessment of a fire on a lowland raised bog in Co. Offaly, Ireland (All Saints Bog). Three burn severities were identified in the field (light, moderate, and deeply burned), and surface charcoals were taken from 17 sites across all burn severities. Charcoals were classified into two fuel type categories (either ground or aboveground fuel) and the reflectance of each charcoal particle was measured under oil using reflectance microscopy. Charcoal reflectance shows a positive relationship with charring temperature and as such can be used as a temperature proxy to reconstruct minimum charring temperatures after a fire event. Resulting median reflectance values for ground fuels are 1.09 ± 0.32%Ro_median_, corresponding to estimated minimum charring temperatures of 447°C ± 49°C. In contrast, the median charring temperatures of aboveground fuels were found to be considerably higher, 646°C ± 73°C (3.58 ± 0.77%Ro_median_). A mixed-effects modeling approach was used to demonstrate that the interaction effects of burn severity, as well as ecotope classes, on the charcoal reflectance is small compared to the main effect of fuel type. Our findings reveal that the different fuel types on raised bogs are capable of charring at different temperatures within the same fire, and that the pyrolysis intensity of the fire on All Saints Bog was primarily driven by the fuel types burning, with only a weak association to the burn severity or ecotope classes.

## Introduction

Peatlands cover ~3% (4 × 10^6^ km^2^) of the Earth's land surface, yet their long-term ability to sequester carbon means they play a significant role in moderating atmospheric CO_2_ (Joosten and Clark, 2002; Connolly and Holden, 2013). However, the human exploitation of peatlands, particularly drainage, peat cutting and fires are all threatening this ecosystem (Connolly and Holden, 2013). Drained peatlands in particular are estimated to cover 5 × 10^5^ km^2^ globally (12% of total global peatland area) and emit up to 2 Gt of CO_2_ (including fires) to the atmosphere annually (Joosten, 2010). Drained peatlands are also more vulnerable to fire (Fernandez et al., 2005) as drainage alters the hydrophysical properties of peat, creating a denser surface peat, limiting the surface moisture contents required for *Sphagnum* growth (Sherwood et al., 2013). The lower fuel moisture contents also reduce the amount of energy required for peat ignition (Benscoter et al., 2011), ultimately resulting in higher severity fires than seen in natural pristine peatlands (Turetsky et al., 2011). Peatland drainage has already been shown to increase both the fire frequency and area burned in Indonesia (Hoscilo et al., 2011), and drained peatlands were also thought to be a major contributor to the 2010 wildfires in Russia (Turetsky et al., 2011; Zaidel'Man, 2011). The subsequent ecological damage of fires on peatlands is thought to depend on the intensity, severity and frequency of the burns (Malone and O'Connell, 2009) and it has been hypothesized that sites with lower water tables burn at higher intensity than pristine peatlands (Ronkainen et al., 2013). Yet the relationships between the fuel types being burned, the hydrology, and resulting fire characteristics (intensity and severity) remain poorly understood for peatlands.

**Figure 1:**
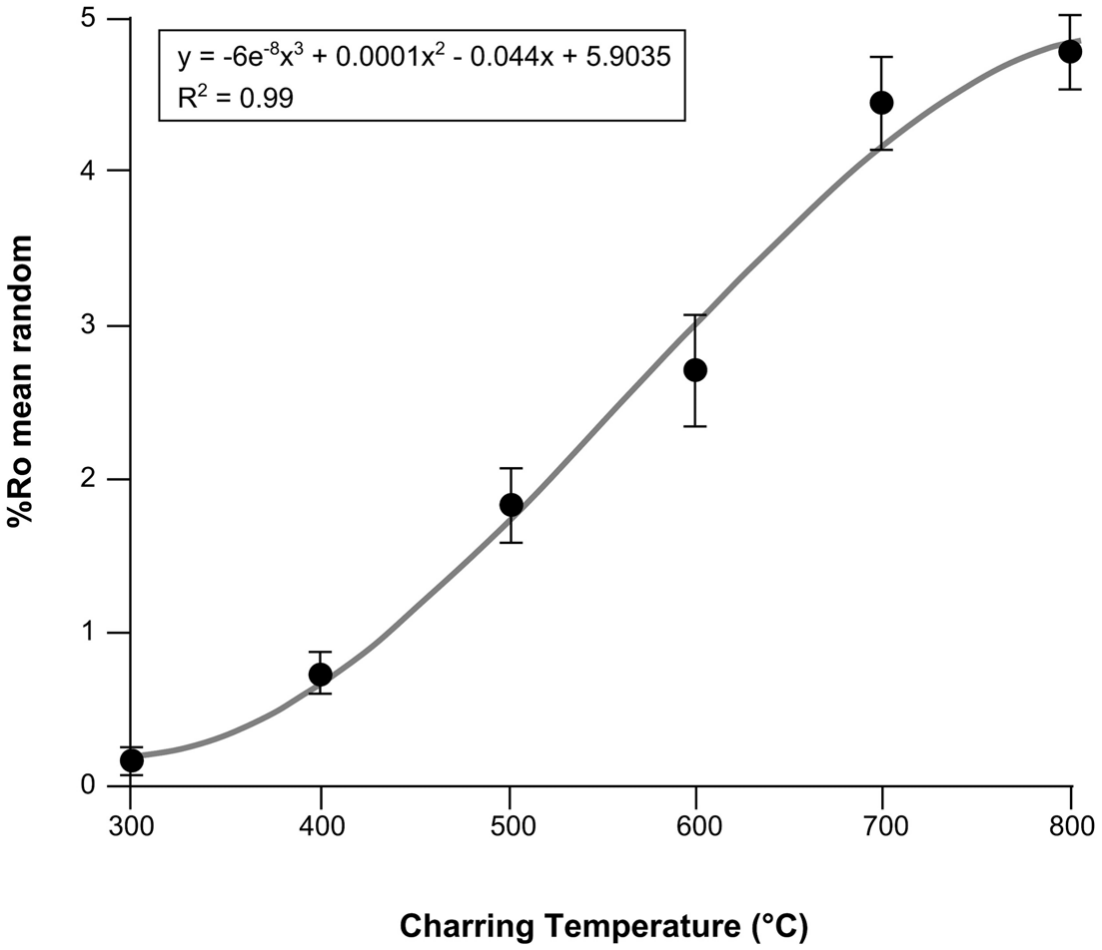
**Combined charcoal reflectance calibration curve for five experimentally charred boreal woods (*Betula nana, Picea mariana, Picea glauca, Betula papyrifera, Populus tremuloides*)**. Mean random reflectance under oil (Ro_mean_) and standard deviations represent all species.

After a fire, a visual assessment of the organic matter loss at the surface (burn severity) can be measured in the field. Yet, there is currently no ground-based, post-fire, quantitative measure of intensity. Fire intensity is traditionally considered as the energy output rate per unit length from the fire front (Alexander, 1982) but, temperature is also considered to be an aspect of fire intensity (e.g., Keeley, 2009), and has been shown to relate to vegetation mortality in other ecosystems (e.g., Elliott et al., 2009). In the absence of direct measures of intensity, it is possible to reconstruct the minimum charring temperatures the plant material was heated to during the wildfire, using reflectance measurements of charcoal. Charcoal is a relatively recalcitrant, slowly cycling organic compound (Ascough et al., 2010) that can be preserved in peats, soils, sediments and rocks for millennia. The molecular structure of charcoal becomes more aromatic with increasing temperature of formation (Preston and Schmidt, 2006). Thus, when studied under oil using reflectance microscopy, this increase in ordering of the charcoal structure translates to a predictable increase in measurable light reflected from the sample (Scott, 2010; Figure 1). This well-established relationship has been used to provide an estimate of formation temperature for charcoal in geological, volcanogenic, and archeological settings (e.g., Jones et al., 1991; Scott and Jones, 1991; Scott and Glasspool, 2005; McParland et al., 2007, 2009), yet this is an underutilized technique for modern wildfire-derived charcoal (e.g., McParland et al., 2009). Charcoal is a product of the thermal decomposition of organic matter in the absence of oxygen (pyrolysis) during combustion events. If combustion proceeds, pyrolysis is then followed by the *in situ* oxidation of the char, as seen in smoldering combustion, resulting in the consumption of charcoal and the formation of ash (Hadden et al., 2013; Rein, 2013). Fires in peatlands may be characterized by smoldering and/or flaming combustion. Smoldering combustion is a slowly propagating, low temperature form of combustion that consumes the organic soils in peatlands. In contrast, shorter duration, higher temperature flaming fires often only pyrolyze the aboveground biomass and litter (Rein et al., 2008, 2009; Hadden et al., 2013). Therefore, we consider charcoal reflectance to represent the minimum temperature plant material is heated to during the pyrolysis stage of combustion, and is herein termed pyrolysis intensity.

A fire in July 2013 on All Saints Bog, a lowland raised bog, in Co. Offaly, provided an opportunity to examine the relationships between, the fuel types being burned, the hydrology, and resulting fire characteristics (pyrolysis intensity and burn severity) on a partially drained peatland. All Saints Bog covers 400 ha, around 30% of which has been impacted by peat extraction (Cole and Mitchell, 2003). It has therefore been suggested that All Saints Bog may be more vulnerable to higher severity burns in the future. Despite cessation of the main industrial peat cutting activity, the expansion of negative indicator species across All Saints Bog suggest that the bog is continuing to dry out (Fernandez et al., 2005). Wetter ecotopes within the bog have a higher tolerance to burning than degraded, drier ecotopes (Cross, 1987). Regular burning could also degrade the microtopography of the bog and cause central ecotopes to become inactive non-peat forming ones (Fernandez et al., 2005) further enhancing the likelihood of fires on this bog. Fires in peatlands are also directly influenced by fuel characteristics such as, fuel availability, loading, composition (fuel type), and condition (bulk density and moisture) (Benscoter et al., 2011). The expansion of drier vegetation types on drained ecotopes of the bog is therefore also likely to alter the fire behavior in the future, yet our understanding of fire behavior in these ecosystems is still limited. In this paper, we provide a post-burn assessment of the 2013 burn on All Saints Bog. Here we examine two factors known to influence fire behavior on drained peatlands, the hydrology (ecotopes) and the fuel types being burned. We assess how these factors relate to the subsequent burn severity and our charcoal reflectance measure of pyrolysis intensity, as a preliminary step in understanding fires on drained peatlands.

## Materials and methods

### Geographic setting, vegetation, and fire history

All Saints Bog is a lowland raised bog with a section of birch bog woodland. The bog is located ~8 km northwest of Birr, Co. Offaly (53°09′10″ N, 7°58′30” W) at an altitude of 40–45 m above sea level (Cole and Mitchell, 2003). Rainfall recorded at Birr Meteorological station (1979–2008) averages 845.7 mm per year. The mean daily temperature ranges from 6.1°C to 13.5°C. All Saints Bog covers 400 ha and around 30% of this area is impacted by peat (turf) extraction (Cole and Mitchell, 2003). The European Community Council Directive 92/43/EEC (Habitats Directive), on the conservation of natural habitats has designated All Saints Bog and Esker an EU Natura 2000 site, a Special Area of Conservation (SAC), and Special Protection Area (SPA), primarily because of the rare patch of birch bog woodland on the site (Fernandez et al., 2005). The *Betula pubescens*, and *Pinus sylvestris* bog woodland is the largest in Ireland (14.34 ha) and is the only extensive stand on a raised bog (Cross, 1987; Kelly et al., 1995; Cross and Lynn, 2013). However, there is evidence for expansion of negative indicator species across All Saints Bog including, *Pinus sylvestris* and *Pteridium*. These all suggest that the bog is continuing to dry out (Cross and Lynn, 2013) despite cessation of the main industrial turf cutting activity, therefore it has been suggested that All Saints Bog may be more vulnerable to higher severity burns in the future.

**Figure 2:**
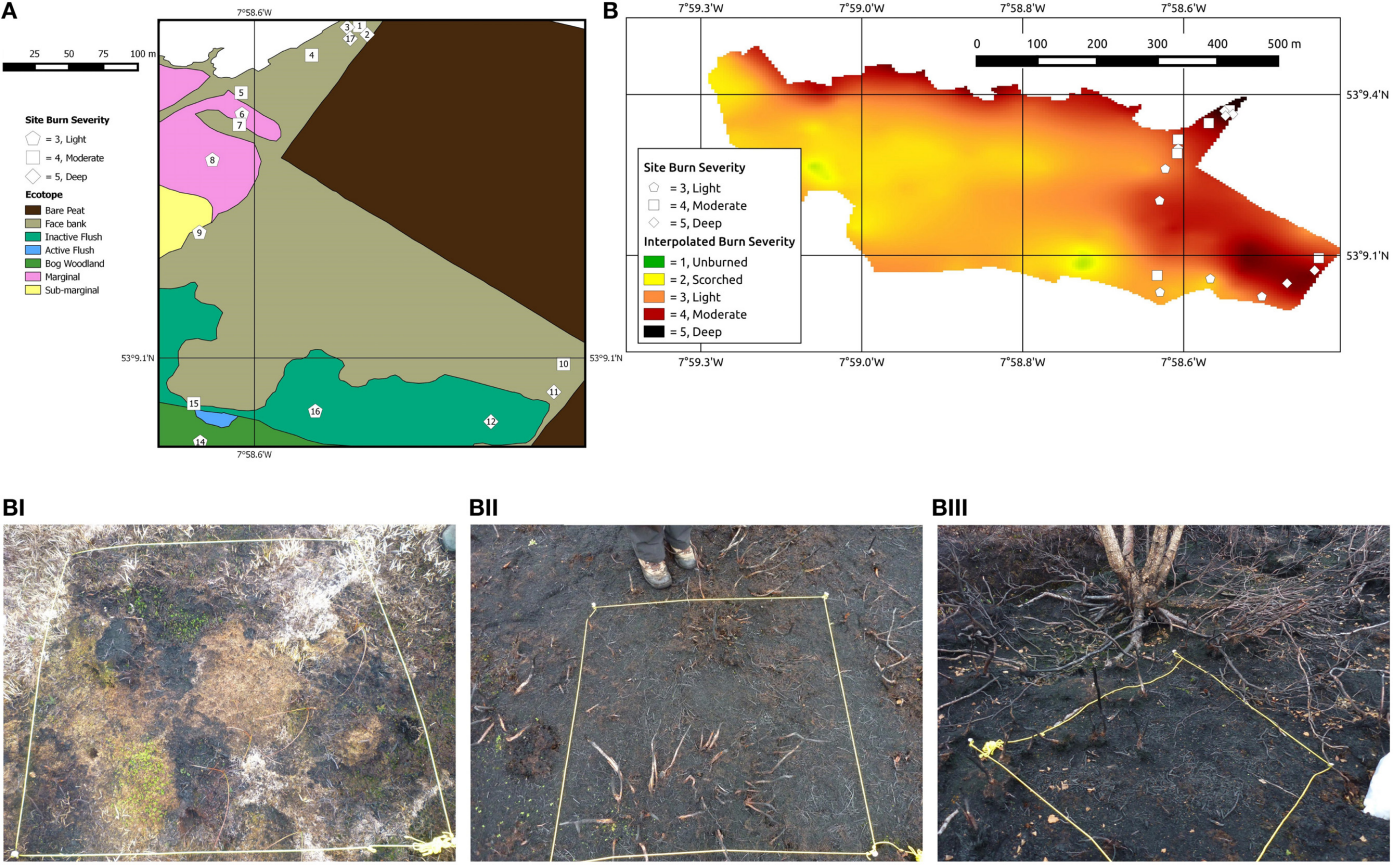
**(A)** Ecotope map of All Saints Bog and position of sampling sites and burn severities (after Fernandez et al., 2012). Ecotopes defined in Table S1. **(B)** Interpolated burn severity map, with sampling localities, illustrating variation in burn severity across the entire 2013 burned area. Visual burn severities were classified based on the Keeley (2009) scheme as modified for raised bog ecosystems (Table 1) and associated field photographs from L-R, (**Bi)** Site 6. Burn severity 3, lightly burned, (**Bii)** Site 10. Burn severity 4, moderately burned, and (**Biii)** Site 11. Burn severity 5, deeply burned.

In July 2013 exceptionally warm and dry conditions [Birr meteorological station average July temperature 22.6°C (max. 28°C) and precipitation (0–25 mm)] provided conditions conducive to burning, and on 14 July a fire burned around 42 ha (~10%) of the bog. The high conservation priority of the bog and bog woodland meant that the fire was extinguished within 4 days. We sampled 17 quadrats across a section of All Saints Bog. Sixteen of these were on the drained, but uncut section of the bog, and one on the bog woodland (site 14) (Figure 2A). Former intensive turf cutting and drainage is a likely driver of increasing fire frequency on All Saints Bog (Fernandez et al., 2005). In 2003, 42% of the active raised bog was burned (Fernandez et al., 2005). The bog also burned in 1991–1992 along the N and W sides (Kelly et al., 1995). As a result of this high fire frequency, post-fire changes in vegetation have been observed in these areas, with a decrease in *Cladonia portentosa* cover as well as an increase in sedges (e.g., *Trichophorum* and *Carex panacea*) and shrubs (e.g., *Calluna vulgaris*) (Kelly et al., 1995).

**Table 1:** **Burn severity field classification after Keeley (2009), severity scores, and modifications for All Saints Bog**.

**Fire severity**	**Description**	**Burn severity score**	**Burn severity classification modifications for All Saints raised bog**	**Figure references**
Unburned	Plant parts green and unaltered, no direct effect from heat	1	Plant parts green and unaltered, no direct effect from heat	Not illustrated
Scorched	Unburned but plants exhibit leaf loss from radiated heat	2	Vegetation on hummocks intact and grasses unaffected, but consumption of fine fuels in the shrub layer	Not illustrated
Light	Surface litter, mosses and herbs charred or consumed. Soil organic layer largely intact and charring limited to a few mm depth	3	Hummocks composed of sedge, *Sphagnum* and lichens killed by radiated heat, but uncharred. Fine fuel from shrub layer consumed (foliage and twigs) some larger stems scorched/partially charred.	Site 6. **Figure 2Bi**
Moderate or severe surface burn	All understory plants charred or consumed. Fine dead twigs on soil surface consumed. Pre-fire soil organic layer largely consumed	4	Understory shrubs (primarily *Calluna*) consumed. Charred bryophyte ground layer and surface peat	Site 10. **Figure 2Bii**
Deep burning or crown fire	Surface litter of all sizes and soil organic layer largely consumed. White ash deposition and charred organic matter to several cm depth	5	Exposed tree roots and charred peat surfaces. Charred and/or consumed shrub layer and bryophyte ground layer.	Site 11. **Figure 2Biii**

#### Ecotopes of all saints bog

Ecotopes are classified based on the microtopography, hydrological conditions, and vegetation community of a given area of the bog (Van der Schaaf and Streefkerk, 2002). All Saints Bog has been classified into eight ecotopes (Fernandez et al., 2012; Figure 2A; Table S1), but charcoal samples were only obtained from five of these: bog woodland (site 14), sub-marginal (site 9), marginal (sites 6–8), face-bank (sites 1–5, 10, 11, 17) and inactive flush (sites 12, 13, 15, 16) (Figure 2A). The majority of All Saints Bog is classified as a degraded raised bog, which in this study encompasses the sub-marginal, marginal, face bank and inactive flush ecotopes (Table S1; Fernandez et al., 2012; Regan et al., 2013), or 16 of the sites studied (Figure 2A).

### Field determinations of burn severity and charcoal sampling

Burn severity mapping by JMY in August 2013 (Figure 2B) revealed a range of burn severities from unburned to deeply burned using the Keeley (2009) classification scheme, modified for a raised bog ecosystem (Table 1). Surficial charcoal samples were collected by VH, CMB, and JMY from 1 m^2^ quadrats for 17 sites across the drained, but uncut area of the peat bog (*n* = 16) and the patch of bog woodland (*n* = 1; site 14; Figure 2A), specifically targeting the area of the bog most affected by the burn (Figure 2B). Sites were selected to encompass the three burn severities (labeled 3–5 in Figure 2B; illustrated in Figures 2Bi–iii; Table S2) and to represent the best approximation of a given burn severity class in that area of the bog (Figure 2B). Of these, six sites were lightly burned (burn severity 3; sites 6, 8, 9, 13, 14, 16; Figures 2B,Bi), six were moderately burned (burn severity 4; sites 1, 4, 5, 7, 10, 15; Figures 2B,Bii), and five were deeply burned (burn severity 5; sites 2, 3, 11, 12, 17; Figures 2B,2Biii; Table S2). Four 200 mL pots of charcoal were handpicked across the surface of each 1 m^2^ quadrat (taking care to sample all charcoal size fractions) and each quadrat was photographed in the field prior to sampling. Field photographs of each quadrat were later loaded into a drawing package (Inkscape v. 0.48) and overlain by a grid. Each intersection was counted, totaling 100 points per quadrat, to give an estimate of macroscopic charcoal cover per quadrat (Table S2).

### Polished block preparation and reflectance microscopy

Charcoal samples were weighed, then dried at 30°C for >90 h, and reweighed. A subsample of charcoal from each quadrat site was handpicked and larger charcoals were cut with a razorblade to ensure that the maximum number of individual particles could be mounted in a single block. Charcoals (four blocks per site) were embedded in polyester resin, and polished by VH and S. Pendray at the University of Exeter, Penryn.

**Figure 3:**
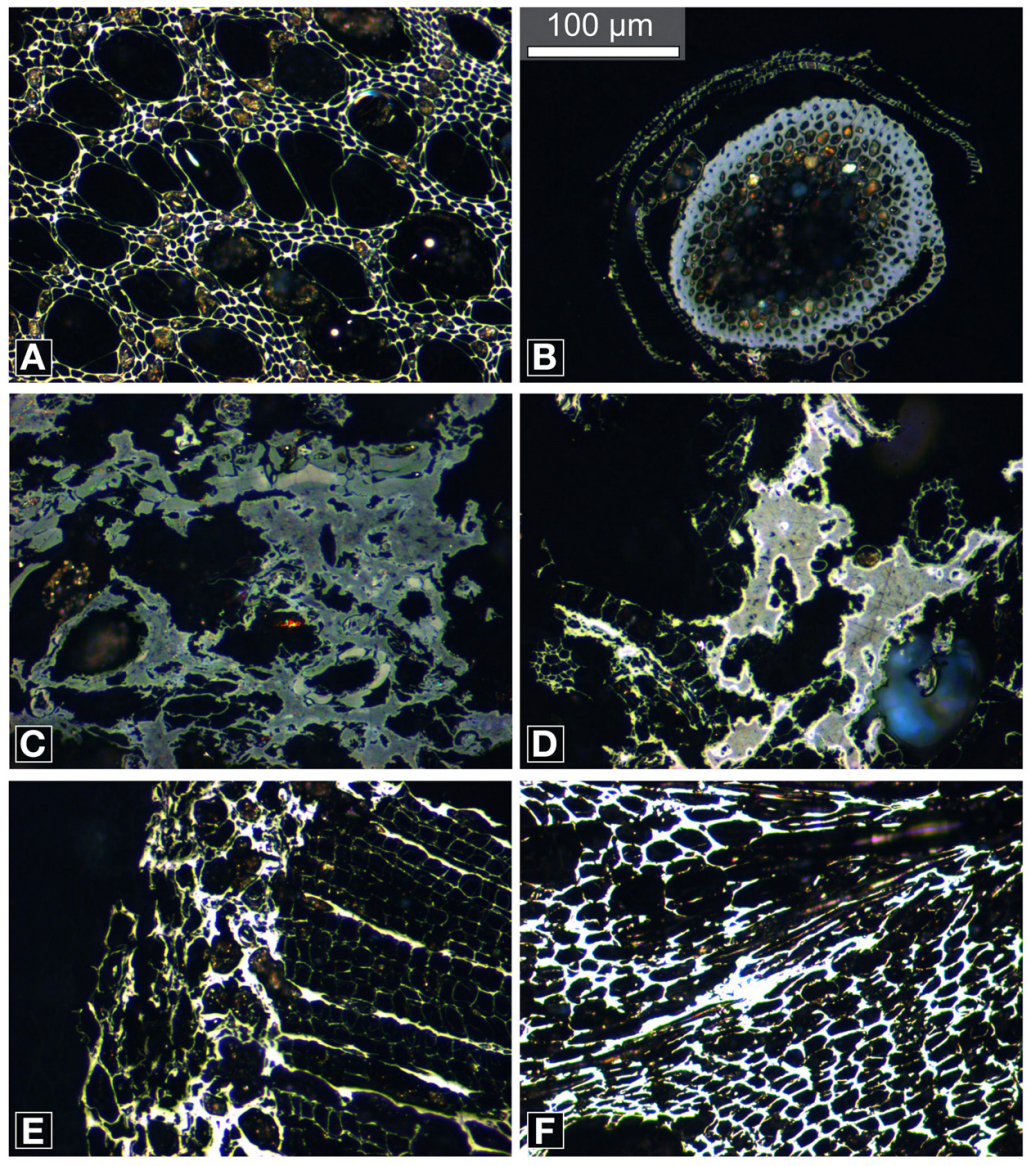
**Reflected-light micrographs of charcoals illustrating the different fuel types observed (A) angiosperm wood, (B) bryophyte, (C) peat clast, (D) peat clast showing high reflecting oxidation rims, (E) gymnosperm wood, and (F) cones**. Note the range in qualitative reflectance from low (gray) to high (white). Scale bar in **(B)** is the same for all images.

The polished blocks were then studied under oil of RI 1.514 at 23°C, using a Leica DM2500P reflectance microscope, with a TIDAS MSP operating system, at Southern Illinois University Carbondale, USA. Under reflected-light (×200 magnification) the charcoals in each sample could be coarsely identified into four groups, angiosperm wood (Figure 3A), bryophytes (Figure 3B), peat (Figures 3C,D), gymnospermous wood (Figure 3E) and cones (Figure 3F). Images were taken using a Leica DFC 400 digital camera. Reflectance measurements were taken at ×500 magnification and were obtained manually using MSP200 v 3.20 software. Reflectance is a photometric measurement of the amount of incident light that is reflected from the polished surface of a charcoal sample when studied under oil (Ascough et al., 2010). The light source used has a wavelength of 546 nm as the photometer is more sensitive near the center of the visible light spectrum (Craig and Vaughan, 1994). Anisotropic organic material, and minerals, can exhibit a range of reflectance values as a function of the crystallographic orientation that is studied (Craig and Vaughan, 1994). The reflectance values reported herein therefore represent random reflectance (%Ro) values as the measurement is taken of the particle at the orientation in which it is encountered (Suárez-Ruiz, 2012). This measurement is then compared to the amount of light reflected by a synthetic standard of known reflectance (Suárez-Ruiz, 2012). The system was calibrated using the following synthetic standards, Strontium Titanite (5.460%Ro), Cubic Zirconia (3.170%Ro), GGG (1.717%Ro), and Glass (0.940%Ro). Ten measurements were made per particle and where possible 30 particles were measured per block, 8391 measurements were taken in total.

### Using charcoal reflectance as a temperature proxy

The study of charcoals (under oil) using reflectance microscopy, enables the formation temperature to be estimated, as the molecular structure of charcoal becomes more aromatic with increasing temperature of formation (Preston and Schmidt, 2006), and this increase in ordering of the charcoal structure translates to a predictable increase in measurable light reflected from the sample (Scott, 2010). This well-established relationship derives from experimentally producing charcoals at known temperatures and for known durations in order to generate calibration curves (i.e., Figure 1). Reflectance values of charcoal produced at unknown temperatures (i.e., wildfire charcoal) can then be extrapolated from these curves, providing an estimate of original formation temperature (Jones et al., 1991; Scott and Jones, 1991; Scott and Glasspool, 2005; McParland et al., 2007, 2009).

#### Charcoal reflectance calibration curve

There is currently no charcoal reflectance calibration curve for peat-forming vegetation, or for the four fuel types studied here. However, experimental charcoalification of a range of plant material including ferns (McParland et al., 2007), fungi (Scott and Glasspool, 2007), and wood (Scott and Glasspool, 2005; McParland et al., 2009; Ascough et al., 2010) have all shown a positive relationship between mean charcoal reflectance and formation temperature.

Fresh wood samples of *Populus tremuloides* (trembling aspen), *Betula nana* (dwarf birch), *Betula papyrifera* (paper birch), *Picea mariana* (black spruce), and *Picea glauca* (white spruce) were cut into 15 mm sized pieces (bark intact), wrapped in foil and heated in steel containers (e.g., Scott and Glasspool, 2005; McParland et al., 2009) in a Carbolite furnace at the University of Exeter. The wood samples were heated at 100°C temperature increments from 300°C to 800°C, each for 1 h duration, to replicate the likely range of temperatures experienced in wildfires (Hudspith et al., submitted). The resulting charcoals were embedded in epoxy resin and polished. Three replicates were analyzed per temperature and 100 random reflectance measurements were taken across the late wood cells in each sample. The data from the five tree species were then combined to generate the calibration curve in Figure 1. This curve demonstrates that charcoal reflectance is strongly correlated with production temperature (*R*^2^ = 0.99; Hudspith et al., submitted) following the polynomial function:

(1) $$y=-6.0\times 10^{-8}x^{3}+1.0\times 10^{-4}x^{2}-4.4\times 10^{-2}x+5.9$$

Where *y* is the formation temperature (°C) and *x* is the charcoal reflectance value (%Ro). Minimum charring temperature (pyrolysis intensity) estimates for fuel types from All Saints Bog are therefore based on the calibration curve in Figure 1. However, charcoal reflectance can be affected by the charring duration. Charcoals produced at <450°C have been shown to attain maximum reflectance after 1 h whereas higher temperature charcoals show a slow increase in reflectance with time, stabilizing after 24 h (Scott and Glasspool, 2005; McParland et al., 2009). We consider the wildfire derived charcoals in this study to have been produced during the pyrolysis stage of flaming combustion during the fire on All Saints Bog. Charring duration over a given area during flaming combustion in a wildfire is likely to be of short duration compared to smoldering combustion (Davies et al., 2013). Therefore, a 1 h calibration curve is valid for interpreting the charring temperatures of the All Saints Bog charcoals. Yet, as the charring duration (post-fire) represents an unknown, and flame lengths, therefore heating, can be variable during flaming combustion (Alexander and Cruz, 2012) the interpreted charring temperatures will be referred to as minimum charring temperatures.

### Identifying fuel types by reflected-light microscopy

Angiosperm wood charcoal (55% of all reflectance measurements) and bryophyte charcoal (31% of all measurements) were observed in all samples at all sites. Charred peat was found in samples from thirteen sites (12% of all measurements) and charred gymnospermous wood and cones at three sites (1% of all measurements) (Figure 3).

#### Angiosperm wood charcoal

Angiosperm wood charcoal was identified based on the presence of vessels (Figure 3A). The family identification of angiosperm woods is complex and the sections through the charcoal studied did not permit identification. However, *Calluna vulgaris* was the dominant shrub species at all sites. It is therefore likely that the majority of angiosperm wood charcoals represent fragments of charred *Calluna*.

#### Bryophyte charcoal

The bryophyte charcoals were categorized based on their characteristic morphology and the presence of a differentiated epidermal layer, cortex, and a central strand composed of hydroids (Figure 3B). Bryophytes occurred as isolated entities, not associated with other plant tissue or matrix, and are therefore not charred peat. Distinction between bryophyte spp. was not possible given that the majority of the bryophyte charcoals measured were isolated traverse sections through stems, and not associated with leaves.

#### Charred peat

Charred peat clasts (this study) are composed of different plant tissues (primarily *Sphagnum* spp.) that have undergone varying stages of degradation prior to charcoalification. These more structured plant tissue components are supported by a matrix of undifferentiated, humified plant tissue of comparable or lower reflectance (Figure 3C). These characteristic features have also been observed in microtome slides of modern peats by Cohen (1973), Cohen and Spackman (1980), Cohen et al. (1999), as well as VH pers. obs. of experimentally charred peat.

#### Charred gymnosperm wood and cones

Gymnosperm wood charcoal (Figure 3E) was rare across all sites. Gymnospermous wood was identified by the presence of repeating rows of similar sized rectangular cells of a single size class with thick cell walls (tracheids) and thin walled cells between (ray parenchyma cells). Cones were only measured at site 2 (part of a cone scale is illustrated in Figure 3F).

### Statistical analysis

The distribution of previously published, wildfire-derived charcoal reflectance values are typically non-normal, with a generally low spread of values (e.g., Scott and Jones, 1994; Scott et al., 2000; McParland et al., 2009). The median is a more robust measure of central tendency for non-normally distributed data. The charcoal reflectance data for the different fuel types measured from All Saints Bog have unequal distributions; therefore the median is reported ± median absolute deviation (MAD) (Tables 2, 3; Table S2), as the MAD is a robust statistic more resilient to outliers than the standard deviation.

**Table 2:** **Summary of the reflectance data for each fuel type and burn severity class**.

**Burn severity class**	**Ground fuels**				**Aboveground fuels**			
	**Bryophytes**		**Peat**		**Angiosperm wood**		**Gymnosperm wood and cones**	
	**% of all Ro values (n)**	**%Ro_median_ (±MAD)**	**% of all Ro values (n)**	**%Ro_median_ (±MAD)**	**% of all Ro values (n)**	**%Ro_median_**	**% of all Ro values (n)**	**%Ro_median (±MAD)_**
3	37 (*n* = 880)	0.96 (±0.56)	9 (*n* = 210)	1.03 (±0.51)	54 (*n* = 1279)	3.63 (±1.01)	0	
4	45 (*n* = 1466)	1.12 (±0.42)	12 (*n* = 380)	0.98 (±0.57)	44 (*n* = 1439)	4.03 (±0.99)	0	
5	13 (*n* = 420)	1.29 (±0.35)	17 (*n* = 560)	1.07 (±0.41)	67 (*n* = 2177)	3.23 (±1.21)	2 (*n* = 80)	2.68 (±1.63)

*For each fuel type, the distribution of data across the burn severity classes (percentages of all reflectance values measured and the number of measurements n), along with the median (%Ro_median_) and median absolute deviations (MAD) for random reflectance under oil*.

**Table 3:** **Summary of the data for each fuel type and ecotope designation**.

**Ecotope**	**Ground fuels**				**Aboveground fuels**			
	**Bryophytes**		**Peat**		**Angiosperm wood**		**Gymnosperm wood and cones**	
	**% of all Ro values (n)**	**%Ro_median (±MAD)_**	**% of all Ro values (n)**	**%Ro_median (±MAD)_**	**% of all Ro values (n)**	**%Ro_median (±MAD)_**	**% of all Ro values (n)**	**%Ro_median (±MAD)_**
Bog woodland	6 (*n* = 20)	1.13 (±0.21)	6 (*n* = 20)	1.27 (±0.14)	88 (*n* = 299)	3.5 (±0.65)		
Submarginal	41 (*n* = 210)	1.23 (±0.26)	12 (*n* = 60)	1.32 (±0.18)	47 (*n* = 240)	3.93 (±0.61)		
Marginal	41 (*n* = 630)	0.98 (±0.4)	17 (*n* = 260)	0.86 (±0.35)	41 (*n* = 630)	3.98 (±0.78)		
Face-bank	33 (*n* = 1656)	1.15 (±0.27)	15 (*n* = 760)	1.03 (±0.3)	50 (*n* = 2456)	3.45 (±0.95)	2 (*n* = 80)	2.68 (±1.1)
Inactive flush	22 (*n* = 530)	0.93 (±0.38)	5 (*n* = 110)	0.99 (±0.23)	73 (*n* = 1770)	3.58 (±0.58)		

*For each fuel type, the distribution of data across the ecotopes (percentages of all reflectance values measured and the number of measurements n), along with the median (%Ro_median_) and median absolute deviations (MAD) for random reflectance under oil*.

Statistical analysis was conducted by JMY, using R software (version 3.1.1) (R Core Team, 2013). The distribution of reflectance values for each fuel type were visualized using a kernel density estimate with a Gaussian window, using a default bandwidth algorithm (nrd0).

We fitted the following linear mixed model to the charcoal reflectance data:

$$\begin{array}{l} {\text{R}}_{\text{ijkx}}\sim \beta_{\text{F}}{\text{F}}_{\text{i}}+\beta_{\text{S}}{\text{S}}_{\text{j}}+\beta_{\text{E}}{\text{E}}_{\text{k}}+\beta_{\text{FxS}}{\text{F}}_{\text{i}}{\text{xS}}_{\text{j}}+\beta_{\text{FxE}}{\text{F}}_{\text{i}}{\text{xE}}_{\text{k}}+\beta_{\text{ExS}}{\text{E}}_{\text{k}}{\text{xS}}_{\text{j}}\\ \;+{\text{b}}_{\text{s}(\text{x})}+\varepsilon_{\text{ijkx}} \end{array}$$

With:

$${\text{b}}_{\text{s}(\text{x})}\sim \text{N}(0,\tau^{2})\text{ and }\varepsilon_{\text{ijkx}}\sim \text{N}(0,\sigma_{\text{i}}^{2})$$

Fuel types, F_i_, were grouped into ground fuels (peat and bryophytes) and aboveground fuels (angiosperm and gymnosperm wood). In the model, F_i_ is the ith fuel type (ground, aboveground), S_j_ is the jth burn severity class (light, moderate, deep), E_k_ is the kth ecotope (marginal, inactive flush, facebank), R_ijkx_ is the reflectance value of the xth data point (corresponding to fuel type F_i_, burn severity S_j_ and ecotope E_k_), β _F_, β_S_ and β_E_ are the main effects of fuel type, burn severity and ecotope respectively. β _FxS_ is the interaction of fuel type and burn severity (other interaction terms are β _FxE_ and β _ExS_), b_s(x)_ is a random effect of site (s(x) is the site from which the xth data point was collected), with a normal distribution and a variance between sites of τ ^2^, and ε_ijkx_ is the residual of the xth data point. The residuals had a normal distribution with a variance that was allowed to depend upon the fuel type class (variance of residuals for the ith fuel type class is σ^2^_i_) because preliminary analysis suggested that reflectance values of aboveground fuels had a higher variance than reflectance values of ground fuels. Reflectance values were obtained for only one site within the bog woodland and submarginal ecotopes and therefore neither of these ecotopes were included in the model. The final model was fitted by maximum likelihood using the nlme package in R (Pinheiro et al., 2014) and the marginal effects of each term (β _F_ = 0, β _S_ = 0, β_E_ = 0, β _FxS_ = 0, β_FxE_ = 0, β_ExS_ = 0) were tested using *F*-tests. Marginal reflectances were used to visualize the results. The marginal reflectance for ecotopes and fuel type were calculated by refitting the model with all terms involving ecotope or fuel type removed, using this model fit to generate the Best Linear Unbiased Predictions (BLUP) for reflectance and subtracting these from the observed reflectances.

## Results

### Charcoal abundance and burn severities in the field

Three burn severities were identified in the field, light (3), moderately (4), and deeply (5) burned sites (Figure 2B). All moderately and deeply burned sites (*n* = 11; sites 1–5, 7, 10–12, 15, 17; Figure 2B) were located in degraded ecotopes (marginal, inactive flush, and face-bank; Figure 2A), likely to be the driest areas of the bog (Table S1). These areas also typically had a dense understory shrub layer (*Calluna vulgaris*) and/or trees (Figure 2Biii). The treed areas that were sampled in this study (*n* = 8; sites 1–3, 11, 12, 14, 16, 17) either occurred as isolated stands of *Betula* spp., or mixed stands of *Betula* spp. and *Pinus* sp. in the densely drained section of the bog. The fire did not crown in the wooded section of the bog and the cones observed at site 2 were charred at ground level. The bark was scorched, but remained attached to the trees. The shrub and moss layers were the most affected by the fire at the moderately and deeply burned sites. Understory bracken had been killed by the radiated heat from the fire, but remained uncharred. Despite the underrepresentation of bracken and tree bark in the resulting charcoal assemblage, quadrats from sites that burned moderately or deeply (*n* = 11) contained the highest percentages of charcoal (burn severity 4 mean = 56%; burn severity 5 mean = 61%; Table S2).

The lightly burned sites (*n* = 6; Figure 2Bi) were from marginal, sub-marginal, and inactive flush (sites 6, 8, 9, 13, 16), as well as, bog woodland (site 14) ecotopes (Figure 2A). Most lightly burned areas were in open hummock/hollow and lawn sections of the bog. Vegetation in the hollows and the shrub layer were generally consumed, yet the hummocks of *Sphagnum*, lichens, and sedges (Figure 2Bi) typically remained intact. Plant tissue mortality occurs at low temperatures (>40–70°C) (Ryan, 2002) and much of the vegetation on hummocks appeared to have been killed by the radiant heat from the fire, but had not been charred (Figure 2Bi). A greater coverage of uncharred material resulted in lower charcoal percentages in the quadrats from the lightly burned sites (mean = 37%; Table S2).

### Charcoal reflectance between fuel types and severities

**Figure 4:**
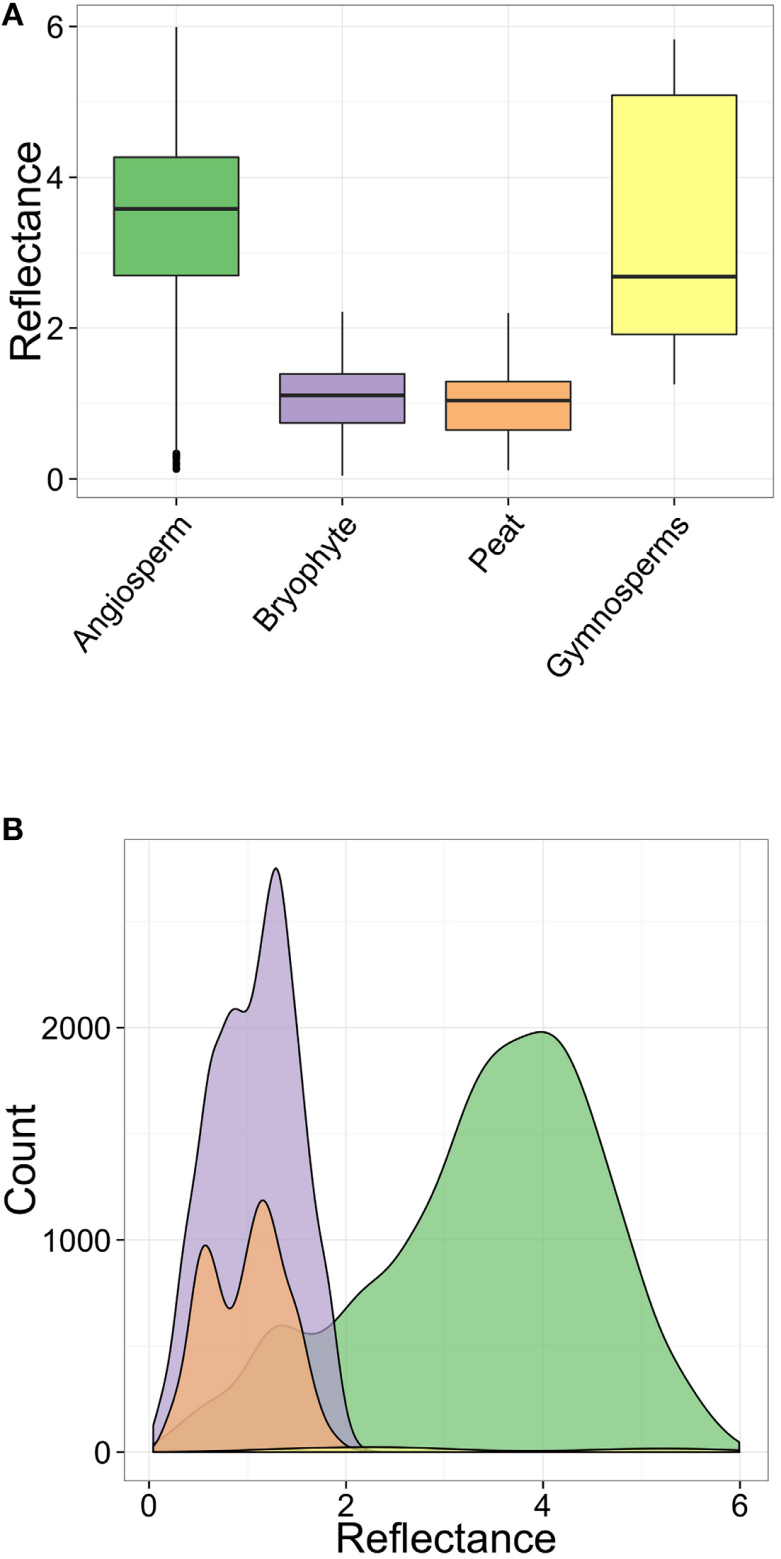
**Charcoal reflectance values for the different fuel types, across all sites and burn severities. (A)** Box and whisker plot. For each fuel type, the box limits are the 25 and 75% quartiles, the central line inside each box is the median and the whiskers are 1.58 times the inter-quartile range. **(B)** non-stacked Kernel density estimate for all data.

The distribution of the kernel density estimate for all charcoal reflectance measurements from All Saints Bog is non-symmetric and bimodal, with a wide spread of data (0.04–5.99%Ro) (Figure 4B). When these data are separated into their respective fuel types, the charcoals identified as bryophytes (*n* = 2266; Figure 3B) ranged from 0.04 to 2.22%Ro (%Ro_median_ 1.11 ± 0.48) (Figure 4; Table 2). Using the polynomial interpolation from the calibration curve in Figure 1, this translates to an estimated range of minimum charring temperatures from <300°C (uncharred) to 540°C (median 447°C ± 47°C). The other ground fuel type, charred peat (*n* = 1150; Figures 3C,D), shows a similar range of reflectance values from 0.11 to 2.2%Ro (%Ro_median_ 1.04 ± 0.48) (Figure 4; Table 2), translating to a comparable range of minimum charring temperatures from <300°C (uncharred) to 540°C (median 440°C ± 49°C).

**Figure 5:**
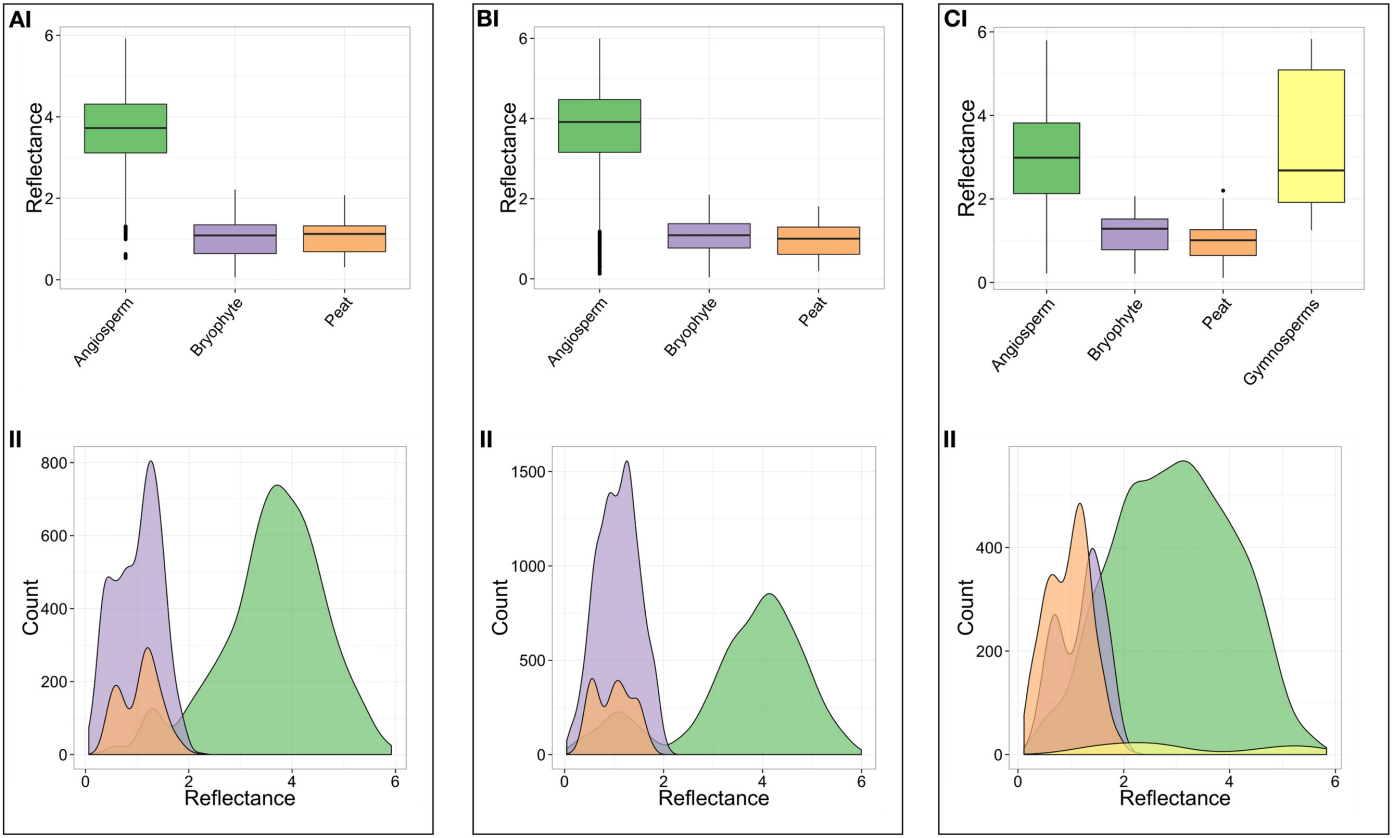
**Charcoal reflectance values grouped according to burn severity (i) Box and whisker plot**. For each fuel type, the box limits are the 25 and 75% quartiles, the central line inside each box is the median and the whiskers are 1.58 times the inter-quartile range. (ii) non-stacked Kernel density estimate for all charcoal reflectance data. **(A)** Light burn, severity 3. **(B)** Moderate burn, severity 4. **(C)** Deep burn, severity 5.

In contrast, aboveground fuels such as angiosperm wood charcoal (*n* = 4895; Figure 3A) range in reflectance from 0.13 to 5.99%Ro (%Ro_median_ 3.58 ± 1.12) (Figure 4; Table 2), with a range in estimated minimum charring temperature from <300°C (uncharred) to >800°C (median 646°C ± 104°C). Gymnosperm wood charcoal (*n* = 50; Figure 3E) and charred cones (*n* = 30; Figure 3F) were extremely rare given the number of treed sites studied (*n* = 8; sites 1–3, 11, 12, 14, 16, 17) and were only observed in samples from deeply burned sites (2, 3, 11) (Table S2; Figure 5C). Charcoal reflectance values ranged from 1.25 to 5.83%Ro (%Ro_median_ 2.68 ± 1.63) (Figure 4) with estimated minimum charring temperatures ranging from 460°C (uncharred) to >800°C (median 575°C ± 136°C). Charred cones yield the highest reflectance of all fuel types, %Ro_median_ 5.28 ± 0.2 (>800°C).

Median charcoal reflectance for the bryophyte and peat (ground fuel) groups range from %Ro_median_ 0.96–1.29 between burn severity classes (Figures 5Ai–Ci; Table 2) translating to a narrow range of estimated minimum charring temperatures from 433°C to 464°C. In contrast, the median reflectance for the aboveground fuels (angiosperm and gymnosperm wood) range from %Ro_median_ 2.68 to 4.03 between burn severity classes (Figure 5; Table 2) or 575°C–686°C.

### Charcoal reflectance of fuel types between ecotopes

Median charcoal reflectance for the bryophyte and peat (ground fuel) groups range from 0.86 to 1.32 between ecotopes (Table 3) translating to a range of estimated minimum charring temperatures from 422°C to 466°C. The median reflectance for the aboveground fuels (angiosperm and gymnosperm wood) range from 2.68 to 3.98 between ecotopes (Table 3), or 575°C–681°C.

### Association between reflectance, fuel type, burn severity and ecotopes

**Figure 6:**
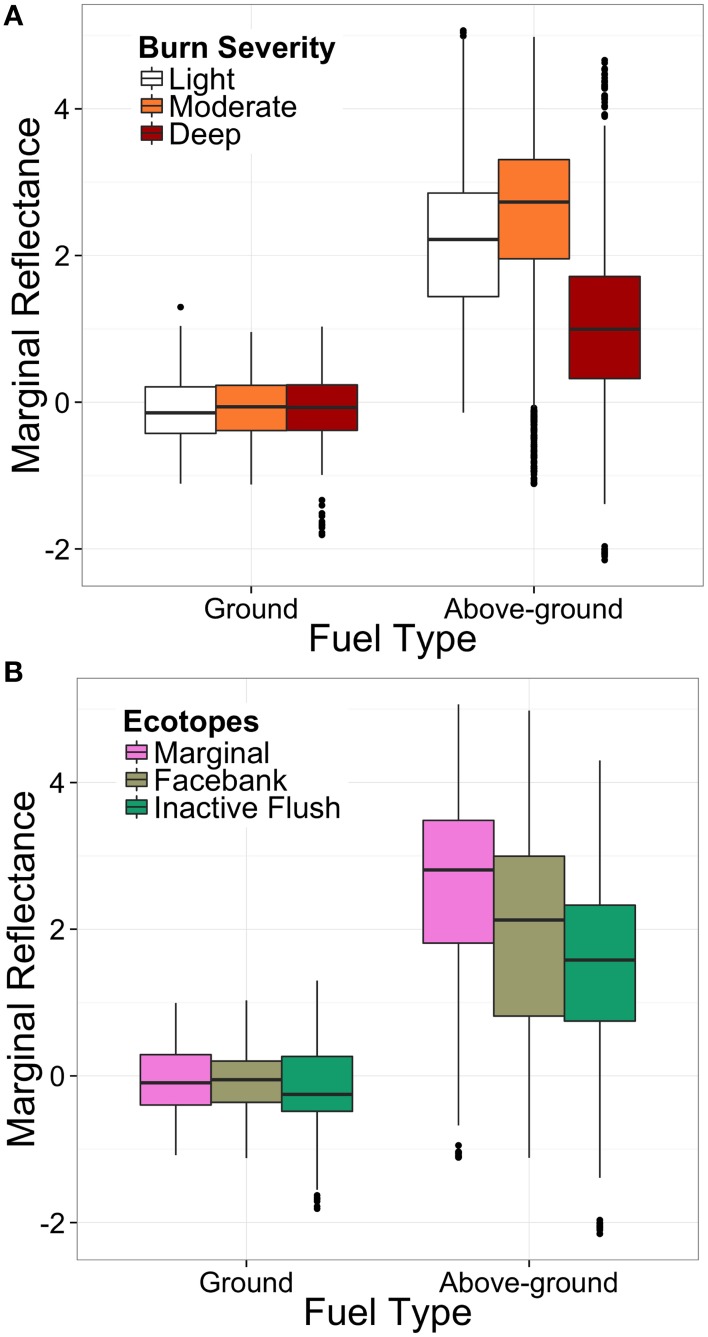
**Comparative box and whisker plots illustrating the marginal effect of (A) fuel type and burn severity and (B) fuel type and ecotope class on charcoal reflectance**. The box limits are the 25 and 75% quartiles, the central line inside each box is the median and the whiskers are 1.58 times the inter-quartile range.

On average, the fuel type being burned represents a strong main effect on the charcoal reflectance [*F*_(1, 8862)_ = 1983.62, *p* ≤ 0.0001]. For lightly burned marginal ecotopes, aboveground fuels are estimated to have a reflectance 2.74 ± 0.06 higher than ground fuels (Table 4). However, the size of the fuel type effect is not completely consistent across burn severities [*F*_(2, 8862)_ = 278.92, *p* ≤ 0.0001] or ecotope categories [*F*_(2, 8862)_ = 5.76, *p* = 0.004], although these interactions are relatively small. This inconsistency in effect sizes can be seen from the difference in marginal reflectance estimates for aboveground fuels between ecotopes (Figure 6B), and the lower marginal reflectance for aboveground fuels for the deepest burned sites (burn severity 5) (Figure 6A; Table 4). Even with these discrepancies, the size of these interaction effects on charcoal reflectance is smaller than the main effect of fuel type (Table 4; Figure 6).

**Table 4:** **Estimated effect sizes and standard errors (SE) for the fixed-effects from the model**.

**Model term**	**Fuel type**	**Severity**	**Ecotope**	**Effect size (SE)**
Main effects	F_1_	S_1_	E_1_	0.85 (0.13)
	F_1_	S_2_-S_1_	E_1_	0.30 (0.18)
	F_1_	S_3_-S_1_	E_1_	−0.03 (0.18)
	F_1_	S_1_	E_2_-E_1_	0.23 (0.18)
	F_1_	S_1_	E_3_-E_1_	0.17 (0.16)
	F_2_-F_1_	S_1_	E_1_	2.74 (0.06)
Interactions	F_2_-F_1_	S_2_-S_1_	E_1_	−0.002 (0.05)
	F_2_-F_1_	S_3_-S_1_	E_1_	−1.06 (0.06)
	F_2_-F_1_	S_1_	E_2_-E_1_	−0.18 (0.05)
	F_2_-F_1_	S_1_	E_3_-E_1_	−0.17 (0.06)
	F_1_	S_2_-S_1_	E_2_-E_1_	−0.38 (0.22)
	F_1_	S_3_-S_1_	E_2_-E_1_	0.18 (0.23)
	F_1_	S_2_-S_1_	E_3_-E_1_	−0.26 (0.24)
	F_1_	S_3_-S_1_	E_3_-E_1_	0.85 (0.24)

*F_*1*_, ground fuel; F_*2*_, aboveground fuel; S_*1*_, burn severity 3; S_*2*_, burn severity 4; S_*3*_, burn severity 5; E_*1*_, marginal ecotope; E_*2*_, facebank ecotope; E_*3*_, inactive flush ecotope*.

In order to visualize the estimated effect each variable (fuel type, severity, ecotope) individually has on the charcoal reflectance, the marginal effects were obtained from the model (and illustrated in Figure 6). The difference in reflectance between ground and aboveground fuels can be observed both between the burn severity (Figure 6A), and ecotope classes (Figure 6B). Despite weakly significant interaction effects between variables, it appears that the charcoal reflectance values measured in this study are primarily driven by the fuel types being burned (Figure 6).

## Discussion

Our post-burn assessment of the 2013 fire on All Saints Bog has demonstrated that the pyrolysis intensity is driven by the fuel types burned at each site (Table 4), with little association between the charcoal reflectance and the burn severity (Table 4; Figure 6A) or ecotope classes (Table 4; Figure 6B). All reflectance values of charcoal identified as ground fuels in this study 1.09 ± 0.32%Ro_median_ are lower than aboveground fuels 3.58 ± 0.77%Ro_median_ (Figure 4A), corresponding to lower estimated minimum charring temperatures for ground (median 447°C ± 49°C) than aboveground fuels 646°C ± 73°C (angiosperm wood median 646°C ± 104°C; gymnosperm wood median 575°C ± 136°C). Therefore, fuels in raised bogs are capable of charring at different temperatures within the same fire, depending on the fuel type. Despite the lack of calibration curves for relevant peat-forming vegetation (resulting in the use of the boreal woods calibration curve in Figure 1), our minimum charring temperature estimates are consistent with other studies of experimentally charred *B. pubescens* wood (780°C) and moss (*Hylocomium splendens*) (450°C) (Van Altena et al., 2012) and consistent in magnitude to field measurements of burning temperatures of *Calluna vulgaris* heathland (680°C–740°C) (Nilsen et al., 2005). Thus, validating our use of the boreal calibration curve, as well as the use of charcoal reflectance as a post-burn estimate of pyrolysis intensity. The differences in pyrolysis intensities between ground and aboveground fuel types on All Saints Bog can be explained by variations in flammability traits such as, fuel moisture content (Benscoter et al., 2011; Davies and Legg, 2011; Santana and Marrs, 2014), bulk density (Benscoter et al., 2011), as well as fuel structure and loading (Benscoter et al., 2011). For example, studies of moorland fires have demonstrated that low fuel moisture is required in both the lower shrub (*Calluna*) canopy and moss/litter layer to enable successful fire spread, and the live fuels in the shrub canopy can even burn independently of the ground fuels (Davies and Legg, 2011). This is because the fine fuels in the shrub layer have a greater propensity to burn in rapidly spreading, high intensity fires, *Calluna* is therefore expected to attain higher charring temperatures during a fire than the ground fuels (moss/peat). This is supported by our results that suggest variations in pyrolysis intensity are related to the individual fuel types burned (Table 5).

**Table 5:** **Results of the mixed-model ANOVA with burn severity, ecotope and fuel type as fixed factors affecting charcoal reflectance**.

	**Numerator DF**	**Denominator DF**	***F*-value**	***p-*Value**
Burn severity, β _S_	2	6	2.04	0.21
Fuel type, β _F_	1	8862	1983.62	<0.0001
Ecotope, β _E_	2	6	0.94	0.44
Burn severity × Fuel type, β _S_ × β _F_	2	8862	278.92	<0.0001
Ecotope × Fuel type, β_E_ × β _F_	2	8862	5.64	0.004
Burn severity × Ecotope, β_S_ × β _E_	4	6	5.76	0.03

*P-values test for the marginal effect of each term*.

The significant interaction between burn severity and fuel type (Table 5), as well as ecotope and fuel type (Table 5), implies that the burn severity and ecotope classes are having some effect on charring temperature; however, this effect is not consistent across fuel types. For example, deeply burned sites show less of a difference in reflectance estimates between ground and aboveground fuels than lightly or moderately burned sites (Figure 6A). This difference translates to a range of estimated minimum charring temperatures from median 617°C ± 103°C (deeply burned) to median 686°C ± 99°C (moderately burned). The lower median charring temperatures for the deeply burned sites may be attributed to differences in botanical affinity of the charcoals within the aboveground fuel category. Median charring temperatures of gymnosperm wood are generally low (575°C ± 136°C), yet gymnosperm wood samples only derive from the deeply burned sites (2, 3, 11), and deeply burned sites in general were typically on treed areas of the bog (sites 2, 3, 11, 12, 17; Figure 2Biii). Moreover, the majority of facebank (5 of 8 sites) and inactive flush (2 of 4 sites) ecotopes were also located on treed areas of the bog and were also characterized by lower aboveground fuel reflectances than marginal ecotopes (0 sites on treed areas) (Figure 6B), meaning that intensities across even the driest areas of the bog appear to be governed by variations in fuel type. Trees observed in the field showed low scorch heights, no evidence of crowning or tree mortality, with charred bark still attached. This may be explained by the difference in fuel particle size between the shrub and tree fuels. Fine fuels (such as *Calluna*) have a greater surface area to volume ratio, as such, ignite more easily, and burn faster than larger fuels (*Betula* branches) during flaming and smoldering combustion (Davies et al., 2008). The lower reflecting, hence lower charring temperature, particles of angiosperm wood at the deeply burned sites may therefore derive from the *Betula* spp. trees, with the higher reflecting particles deriving from the *Calluna vulgaris* shrub layer. These fuel-related variations in charcoal reflectance emphasize the importance of identifying the botanical affinity of the charcoal when interpreting charring temperatures from wildfire-derived charcoal. Charcoal reflectance is a well-established, but currently underutilized technique in estimating charcoal formation temperatures. Previous work has typically reported a single charcoal reflectance value to represent the overall charring temperatures of the entire wildfire-derived charcoal assemblage (e.g., Scott et al., 2000; McParland et al., 2009; Scott, 2010). We have demonstrated that the charcoal reflectance appears to be dependent on the fuel types being burned; therefore, reporting a single value for an entire charcoal assemblage is unlikely to be an accurate representation of the charring temperatures experienced by the different fuel types.

Visual differences between lightly burned (burn severity 3) and deeply burned (burn severity 5) sites were observed in the field (Figures 2Bi–iii). These also appeared to be directly related to localized variation in fuel composition across All Saints Bog, as lightly burned sites were characterized by the presence of uncharred hummocks of moss and lichen (Figure 2Bi). The moss cover likely reduced the organic matter consumption during the fire (Shetler et al., 2008), as hummock-forming *Sphagnum* spp. have high water retention capacities (Benscoter and Wieder, 2003; Fernandez et al., 2005; Shetler et al., 2008), even at low water-table levels (Price and Whittington, 2010). As exemplified by the lower percentage of peat charcoal at lightly burned compared to deeply burned sites (Table 2). In contrast, the deepest burned sites showed evidence for consumption of ground fuels during the fire, as these contained the most charred peat (17%; Table 2) and lowest percentage of bryophyte charcoal (13%; Table 2). However, the presence of charred peat in these samples (illustrated in Figures 3C,D) suggests that only pyrolysis of the peat surface occurred, as the oxidation phase of smoldering combustion of fuel leaves little residue in the form of charcoal (Rein et al., 2008, 2009; Clay and Worrall, 2011; Hadden et al., 2013). There is further evidence for short duration charring of peat as shown by the occurrence of higher reflecting perimeters around some of the charred peat clasts (Figure 3D). Such incomplete charring may be due to localized variations in fuel moisture within the peat, the reduction of oxygen availability at depth (Miyanishi and Johnson, 2002), or quenching of the fire, disrupting charring and preventing peat smoldering, as the fire was extinguished.

The 2013 fire on All Saints Bog was rapidly extinguished before prolonged smoldering of the peat could occur, thus, it was not possible to assess the maximum potential burn severity on the bog. This factor should be taken into consideration when assessing fires on other raised bogs, as dry peats in drained bogs are ordinarily susceptible to high severity, deep burning (Turetsky et al., 2011) due to smoldering of the peat (Page et al., 2002, 2009; Turetsky et al., 2011). The majority of sites studied on All Saints Bog were from degraded ecotopes (Figure 2A), likely the driest areas of the bog and therefore conducive to deep burning, smoldering fires. As the fire was extinguished, this likely limited the maximum potential ecological damage to the bog that would have been experienced if smoldering continued, thus emphasizing the need to actively manage fires in these ecosystems, particularly as degraded raised bogs are more vulnerable to fire than pristine peatlands.

Our charcoal reflectance measure of pyrolysis intensity primarily documents the flaming combustion of the surface and upper ground fuels, and the potential utility of the charcoal reflectance method is still being developed. Nonetheless, our results indicate that charring temperatures can vary in a single fire event as a function of the fuel types burning, and this is something that has not previously been considered in the analysis of wildfire-derived charcoal reflectance in other ecosystems (e.g., Scott et al., 2000; McParland et al., 2009). Further, this is the first use of the charcoal reflectance method in a post-burn assessment of a raised bog, and these data therefore provide additional information about charring temperatures that was previously unknown for raised bog ecosystems.

## Conclusion

The frequency and severity of fires are higher for drained peatlands than pristine peatlands, yet the interactions between bog hydrology, fuel types being burned, pyrolysis intensity, and burn severity are poorly understood for drained peatlands. A detailed post-burn assessment of 17 sites on a partially drained, lowland raised bog in Co. Offaly, Ireland (All Saints Bog) revealed that the minimum charring temperatures were primarily driven by the fuel types being burned, and were only weakly associated with the burn severity or ecotope classes. Although reflectance values are consistently higher for aboveground than ground fuels, associations between burn severity and reflectance, as well as ecotope class and reflectance, were noted; however, the effect sizes were not consistent between burn severity or ecotope classes. This is due to the presence of trees at the most deeply burned sites. These were less fire damaged than the shrub or moss layer, resulting in a charcoal assemblage with a wide distribution of reflectance values for deeply burned sites. In addition, All Saints Bog is a protected site and the fire was rapidly extinguished, therefore extensive smoldering of the peat did not occur, and as a result, it was not possible to assess the potential maximum severity of the 2013 burn. This is the first use of the charcoal reflectance method for measuring pyrolysis intensity in a post-burn assessment of a raised bog and emphasizes the importance of identifying the fuel type prior to making inferences about charring temperature, as the different fuel types in raised bog ecosystems are capable of charring at different temperatures within the same fire event.

## Conflict of interest statement

The authors declare that the research was conducted in the absence of any commercial or financial relationships that could be construed as a potential conflict of interest.
