# Supplementary material for: Charring temperatures are driven by the fuel types burned in a peatland wildfire
Source: Front Plant Sci. 2014 Dec 16;5:714. doi: 10.3389/fpls.2014.00714 (PMC4267186; doi:10.3389/fpls.2014.00714)
Supplement: Supplementary file 1 [file Table1.DOCX]

| **Ecotope class** | **Hydrological Properties** | **Dominant vegetation** |
| --- | --- | --- |
| Bog woodland | Humid-wet peaty substrate. Higher water level than surrounding water table. | *Betula pubescens* dominating the canopy and a ground layer of *Sphagnum* spp. (>25%) and lichens. Thin shrub layer of *Betula* and *Salix* spp. and well-developed dwarf shrub (*Calluna vulgaris*) and herb layers (*Molinia caerulea*, *Juncus effusus* and *Dryopteris erythrosora*). Negative indicator species such as *Pinus sylvestris* and bracken (>10%) indicate dry conditions on the bog. |
| Sub-marginal | Hard to soft surface. Water level 10 cm below the surface. Thin acrotelm, but some differentiation between hummocks and hollows. | Lawns of *Sphagnum* spp. *Trichophorum* common, but in less tussocky form than in the marginal ecotype. *Rhynchospora fusca* occurs in hollows and pools. Hummocks dominated by *Calluna vulgaris*, *Sphagnum*, *Cladonia portentosa*. Hollows dominated by *Nathecium ossifragum* and *Sphagnum.* |
| Marginal | Water level 10 - 40 cm below surface. Some runoff at peak rainfall. Acrotelm absent or poorly developed. Dry surface, degraded microtopography. Hollows can be frequent and dominated by tussocks. Pools rare to absent. | In lawns *Narthecium ossifragum* is most dominant and small patches of *Sphagnum* spp. *Trichophorum* common in tussock form. In small hummocks *Calluna vulgaris* is dominant. |
| Face-bank | Water table 10 - 100 cm below the surface in all seasons. Runoff may occur at peak rainfall. Dry surface, degraded microtopography with low hummocks, hollows and lawns. No pools or wet habitats. | Vegetation usually dominated by *Calluna vulgaris*. |
| Inactive flush | Water movement, but not actively peat-forming. | |

**Table S1** Definitions of the five ecotopes observed in this study (Figure 2 A). Definitions from: Irish Peatland Conservation Council (IPCC) scheme; van der Schaaf and Streefkerk (2002); Fernandez et al. (2012); Cross and Lynn (2013) and Regan et al. (2013).
