# Supplementary material for: Charring temperatures are driven by the fuel types burned in a peatland wildfire
Source: Front Plant Sci. 2014 Dec 16;5:714. doi: 10.3389/fpls.2014.00714 (PMC4267186; doi:10.3389/fpls.2014.00714)
Supplement: Supplementary file 2 [file Table2.DOCX]

| **Locality** | **Burn**  **Severity** | **Quadrat % charcoal cover** | **Ground Fuels** | | | | | | **Aboveground Fuels** | | | | | |
| --- | --- | --- | --- | --- | --- | --- | --- | --- | --- | --- | --- | --- | --- | --- |
|  |  |  | **Bryophytes** | | | **peat** | | | **Angiosperm wood** | | | **Gymnosperm wood/ cones** | | |
|  |  |  | ***%Ro_median_*** | ***MAD*** | ***N*** | ***%Ro_median_*** | ***MAD*** | ***N*** | ***%Ro_median_*** | ***MAD*** | ***N*** | ***%Ro_median_*** | ***MAD*** | ***N*** |
| 1 | 4 | 59 | 1.03 | 0.36 | 100 |  |  |  | 3.33 | 1.87 | 360 |  |  |  |
| 2 | 5 | 64 | 1.32 | 0.48 | 30 | 1.01 | 0.41 | 160 | 2.35 | 0.67 | 320 | 3.57 | 2.30 | 60 |
| 3 | 5 | 44 | 1.36 | 0.36 | 150 | 1.04 | 0.49 | 300 | 2.36 | 1.20 | 210 | 2.78 | 0.28 | 10 |
| 4 | 4 | 63 | 1.03 | 0.39 | 300 | 0.68 | 0.31 | 140 | 4.26 | 0.95 | 229 |  |  |  |
| 5 | 4 | 44 | 1.20 | 0.38 | 317 | 1.10 | 0.43 | 20 | 4.04 | 0.67 | 190 |  |  |  |
| 6 | 3 | 26 | 0.77 | 0.32 | 160 | 0.82 | 0.1 | 50 | 1.33 | 1.11 | 100 |  |  |  |
| 7 | 4 | 64 | 1.33 | 0.48 | 220 | 1.05 | 0.64 | 180 | 4.16 | 0.92 | 440 |  |  |  |
| 8 | 3 | 49 | 0.77 | 0.60 | 250 | 0.65 | 0.6 | 30 | 4.62 | 0.87 | 90 |  |  |  |
| 9 | 3 | 47 | 1.23 | 0.38 | 210 | 1.32 | 0.27 | 60 | 3.93 | 0.9 | 240 |  |  |  |
| 10 | 4 | 61 | 1.06 | 0.4 | 529 | 1.1 | 0.18 | 40 | 4.39 | 0.57 | 220 |  |  |  |
| 11 | 5 | 72 | 1.5 | 0.22 | 70 |  |  |  | 3.1 | 1.39 | 469 | 1.38 | 0.17 | 10 |
| 12 | 5 | 57 | 1.11 | 0.17 | 10 |  |  |  | 3.5 | 0.94 | 720 |  |  |  |
| 13 | 3 | 27 | 1.37 | 0.47 | 50 |  |  |  | 3.66 | 0.84 | 510 |  |  |  |
| 14 | 3 | 40 | 1.13 | 0.31 | 20 | 1.27 | 0.21 | 20 | 3.5 | 0.96 | 299 |  |  |  |
| 15 | 4 | 43 | 0.91 | 0.53 | 280 | 1.06 | 0.19 | 60 | 3.59 | 0.73 | 500 |  |  |  |
| 16 | 3 | 31 | 0.78 | 0.64 | 190 | 0.73 | 0.39 | 50 | 3.26 | 1.39 | 40 |  |  |  |
| 17 | 5 | 68 | 1.14 | 0.25 | 160 | 1.15 | 0.23 | 100 | 3.72 | 0.8 | 458 |  |  |  |

**Table S2** Summary table for each site (quadrat) indicating the observed burn severities (Table 1; Figure 2 B). The percentage of each quadrat covered by macroscopic charcoal (100 points counted per quadrat). %Ro_median_ and median absolute deviations (MAD) of charcoal reflectance measurements, as well as the total number of reflectance measurements taken for each fuel type per quadrat.
